# A Case Report of Malignant Melanoma of the Sphenoid Sinus

**DOI:** 10.1155/2013/613472

**Published:** 2013-06-03

**Authors:** Kiyoaki Tsukahara, Kazuhiro Nakamura, Ray Motohashi, Minoru Endo, Hiroki Sato

**Affiliations:** Department of Otolaryngology, Tokyo Medical University Hachioji Medical Center, 163 Tatemachi, Hachioji, Tokyo 193-0998, Japan

## Abstract

Malignant melanoma of the sphenoid sinus is a very rare disease, and only 6 cases have previously been reported. The present case involved a 74-year-old woman who was examined for visual disturbance of the left eye. Computed tomography revealed a soft tissue shadow, but only mucosal hypertrophy was found on opening the sphenoid sinus under general anesthesia. One month postoperatively, visual disturbance of the right eye and paresis of cranial nerve III appeared. Malignant melanoma was diagnosed from biopsy. Multiple bone metastases were identified, but the patient declined active treatment. As a result, palliative care was provided and she died 3 months later. When there is no improvement in postoperative visual acuity as in this case, in consideration of the possibility of neoplastic lesions, rigorous followup including monitoring for neurological symptoms is warranted.

## 1. Introduction

Almost 20% of melanomas (cutaneous and mucosal) originate in the head and neck, but only 1% arises from the sinonasal tract [1]. Malignant melanomas originating in the sphenoid sinus are extremely rare and only 6 cases have been reported previously [2–7]. We report the seventh case of melanoma arising from the sphenoid sinus.

## 2. Case Presentation

**Figure 1:**
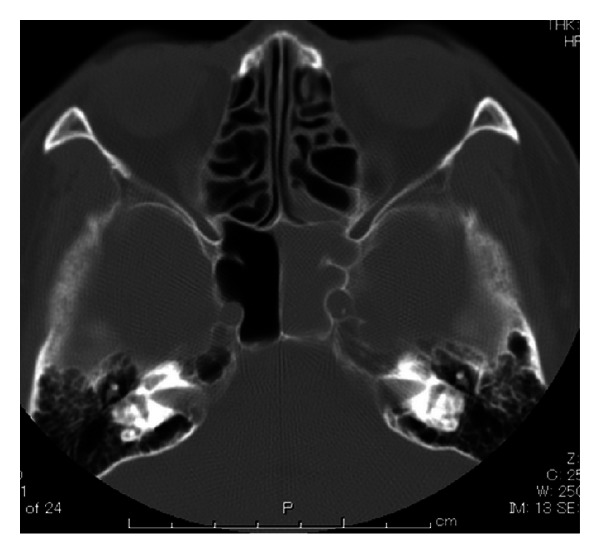
CT findings. The left sphenoid sinus shows a soft tissue shadow without bone damage.

The patient was a 74-year-old woman who was initially examined in neurosurgery for visual disturbance of the left eye. As computed tomography (CT) revealed a shadow in the sphenoid sinus, she was examined in the Department of Otolaryngology of Tokyo Medical University Hachioji Medical Center, revealing a polyp in the middle nasal meatus. Although a soft tissue shadow was observed in the left sphenoid sinus, no damage to the surrounding bone tissue was evident (Figure 1). Preoperative visual acuity was 0.03. No signs of cranial nerve paresis were observed. On the same day, the left sphenoid sinus was opened under general anesthesia using an endoscope. 

**Figure 2:**
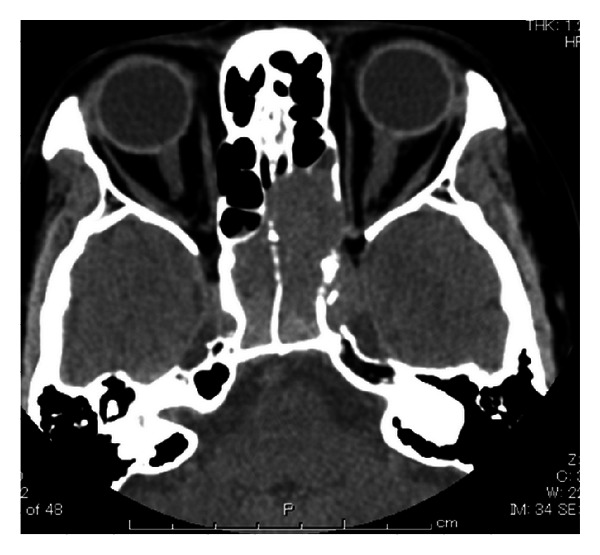
CT findings. In both the left and right sphenoid sinuses, soft tissue shadows accompanying skull base damage are apparent.

**Figure 3:**
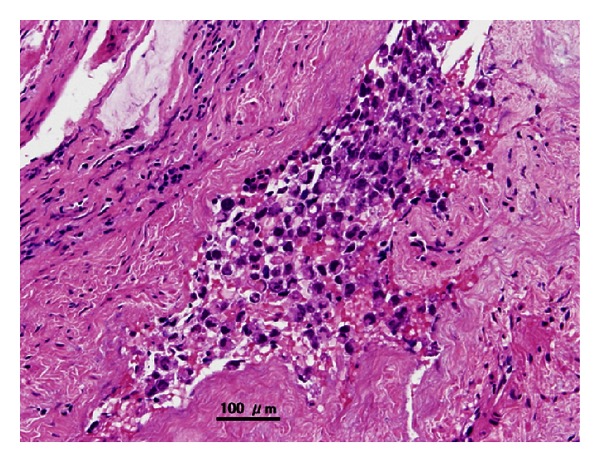
Pathological findings. Amorphous medullary proliferation of medium-sized circular atypical cells is apparent. Nuclei are deeply stained, many are eccentrically located, cytoplasm is lightly eosinophilic, and some cells show brown pigment in parts.

Mucosal hypertrophy was found, and the procedure was completed following removal of the mucosal lesion. Pathology specimens of the removed mucosal lesion were found to represent sinus mucosa with no neoplastic changes. As postoperative visual acuity only improved to 0.6, postoperative steroid therapy was initiated but did not improve vision. One month after surgery, visual disturbance of the right eye, headache, and ophthalmalgia appeared. Visual acuity was negative for light sense, and paresis of cranial nerve III was observed. CT showed soft tissue shadows in both sphenoid sinuses and the left posterior ethmoid sinus, accompanying skull base damage (Figure 2). Using an endoscope, biopsy of the right sphenoid sinus was conducted under general anesthesia. Pathological examination revealed medium-sized, circular atypical cells manifesting amorphous medullary proliferation. Nuclei were deeply stained and frequently eccentrically located, cytoplasm was lightly eosinophilic, and cells with small amounts of brown pigment were evident in parts. Immunohistochemically, the tumor cells were positive for HMB-45 and melan A antibodies, and a very small number were positive for cytokeratin (MNF-116). On the basis of these findings, malignant melanoma was diagnosed (Figure 3). Multiple metastases, to the spine and ischium, were observed on positron emission tomography-CT. As the patient did not desire active treatment such as chemotherapy, only palliative care was provided, and she died 3 months later. 

## 3. Discussion

The paranasal sinus is adjacent to the orbit, and paranasal sinus lesions are known to give rise to ocular manifestations. In cases of visual disturbance due to paranasal sinus mucoceles, visual acuity can be expected to improve if treated within a month of onset [8]. In the sphenoid sinus, however, near the cavernous sinus, malignant cancerous lesions can damage the skull base and progress further. 

In previously reported cases of malignant melanoma of the sphenoid sinus, decreased visual acuity, headache, cranial nerve III paresis, and epistaxis have been frequent initial symptoms [7]. In the present case, no cancerous lesions were evident at the time of the initial procedure, but decreased visual acuity appeared in the right eye, and cranial nerve III paresis developed after a month. Findings at the time of the initial procedure gave no reason to suspect neoplastic lesions, and even from a retrospective examination including CT findings, a correct diagnosis would have been difficult to make at that time. Therefore, in the future, when there is no improvement in postoperative visual acuity, rigorous followup, including monitoring for neurological symptoms, appears warranted.

Given the rarity of malignant melanoma of the sphenoidal sinus, establishing treatment guidelines is difficult, and treatment is therefore made according to the guidelines for sinonasal melanoma [4]. The results of skull base resection for mucosal malignant melanoma have been very poor [9]. Also, while good local control can be obtained with carbon ion radiotherapy, distant metastasis reduces survival rates [10]. However, concomitant use of dacarbazine, nimustine, and vincristine (DAV) with carbon ion radiotherapy has been reported to improve survival rates [11]. In a case like the current one where multiple metastases are present at the time of diagnosis, chemotherapy is normally used, but the response rate of metastatic melanomas to DTIC is only around 15% [12], which is not satisfactory. Although our patient did not desire active treatment and only received palliative care, therapy for this type of disease should be established.
